# Parental separation, negative life events and mental health problems in adolescence

**DOI:** 10.1186/s12889-023-17307-x

**Published:** 2023-11-29

**Authors:** Kateryna Karhina, Tormod Bøe, Mari Hysing, Sondre Aasen Nilsen

**Affiliations:** 1 Department of Psychosocial Science, Faculty of Psychology, University of Bergen, Bergen, Norway; 2 Regional Centre for Child and Youth Mental Health and Child Welfare, NORCE Norwegian Research Centre, Bergen, Norway; 3 Department of Health Promotion and Development, Faculty of Psychology, University of Bergen, Bergen, Norway

**Keywords:** Parental separation, Mental health, Negative life events, Adverse childhood conditions, Adolescence

## Abstract

**Background:**

Parental separation is associated with mental health problems in adolescence. One suggested pathway for this association is through the accumulated exposure to stress and other negative life events. This study aimed to document the distribution of negative life events among adolescents with separated compared to non-separated parents, and to assess the direct and interactive associations between parental separation, negative life events, and mental health problems in adolescence.

**Methods:**

Data stem from the cross-sectional population-based youth@hordaland study of adolescents (aged 16–19) conducted in Norway in 2012, providing self-reported information about parental separation, negative life events, and depression-, anxiety-, conduct-, and ADHD symptoms. Regression analyses were used to assess the direct and interactive associations between parental separation, negative life events, and mental health problems.

**Results:**

Adolescents with separated parents had more mental health problems across all symptom scales compared to peers with non-separated parents, with standardized mean differences [SMDs] ranging from 0.15 to 0.20. Negative life events moderately attenuated these differences (reduced the SMDs with about 0.04–0.08, depending on the outcome). However, none of the interactions between parental separation and negative life events on mental health problems were statistically significant.

**Conclusions:**

Higher exposure to negative life events explains parts of the association between parental separation and mental health problems in adolescence. However, a parental separation does not seem to increase the vulnerability of the effects of negative life events on adolescents’ mental health. Assessing exposure to negative life events is important when providing mental health services to adolescents, particularly to those who have parents separated.

**Supplementary Information:**

The online version contains supplementary material available at 10.1186/s12889-023-17307-x.

## Introduction

Parental separation is associated with more mental health problems in children and adolescents [1]. For example, studies have shown that adolescents with separated parents have a higher risk of emotional and behavioural problems and have a higher prevalence of psychiatric disorders than peers living in non-separated two-parent families [2, 3]. Moreover, these differences are not only confined to childhood but tend to persist into adulthood [4].

One pathway through which parental separation influences children’s mental health is through adverse negative life events. As noted by the divorce-stress-adjustment perspective [5], a parental separation may set into motion several family stressors, such as parental mental health issues, financial problems and family conflicts. Such stressors may also put children at risk of serious negative life events, such as accidents, violence, and death of close ones [6], which are the focus of the current investigation.

A growing body of research has documented that exposure to negative life events is a robust predictor of mental health outcomes extending from childhood through adulthood [7–10]. According to the cumulative risk hypothesis, it is the accumulation of risk, more than the specific types of risk factors, that are important for children and adolescents’ mental health [11, 12]. For example, an early longitudinal study from the U.S. of at-risk children (*N* = 171) found that the number of risk exposures at age 7 strongly predicted both externalizing and internalizing mental health problems at age 16 [13]. More recent work has provided similar findings. Indeed, a recent study drawing on data from the national representative National Survey of Children’s Health in the U.S. (*N* = 29,617), found a graded association between number of negative life events and outcomes such as depression, anxiety, conduct disorder, and ADHD in adolescence [14].

Although the separate associations between parental separation and negative life events with mental health outcomes in adolescence have been well-documented, there is a lack of studies that have examined how parental separation and negative life events jointly influence adolescents’ mental health, and whether negative life events disproportionately affect children with separated parents. Studies often include parental divorce or separation within a cumulative risk framework [13, 15], but few studies have considered whether these factors interact. However, experiencing a parental divorce or separation may not only increase the risk of experiencing negative life events, but may also lower the tolerance levels of future negative events, making it more difficult for children and youth to cope with such events later in life [16]. This may occur due to the emotional distress children may experience, as well as other family issues such as family conflicts and lower parental support and supervision in the post-separation period [17].

Although very little empirical work has considered such interaction effects, one study found a significant interaction effect between parental marital status and childhood abuse, whereby the association between childhood abuse and lifetime ADHD diagnosis was stronger for adolescents who also had experienced parental separation [12]. Another study found that children who had experienced divorce were generally more likely to rate negative life events as more difficult to adjust to, compared to those without divorce experiences [6]. We are, however, unaware of more population-based studies that have examined whether cumulative exposure to negative life events disproportionately affects adolescents with separated parents.

The Norwegian welfare state is characterized by an elaborate social safety net, including free access to health care and family related benefits. Highly subsidized childcare and schools, combined with generous parental leave rights, have led to the dual-earner family being the norm [18]. Moreover, in the case of divorce, custodians are provided support through tax deductions, cash allowances, and child support. Despite these benefits, a parental divorce or separation have been associated with more mental health problems also in Norway, with similar effect sizes to those found in the US [19]. Previous investigations drawing on the same data as the current have also shown that those in separated or single-parent families have more sleep problems [20] and poorer academic outcomes than peers from non-divorced two-parent families [21]. We are, however, unaware of previous Norwegian studies that have investigated the interrelationship between parental separation, negative life events, and mental health outcomes in adolescence.

Given these considerations, this study sought to document the distribution of negative life events among adolescents based on whether their parents had divorced/separated or were living together. We further sought to examine the degree to which exposure to negative life events may account for some of the association between parental separation and mental health outcomes in adolescence, and whether the association between negative life events and mental health problems is moderated by parental separation. Based on existing theory and empirical research, we expected that adolescents with separated parents had a higher exposure to negative life events and more mental health problems than peers with non-separated parents, and that the higher exposure to negative life events would account for some of the association between parental separation and adolescent mental health problems. Due to few previous empirical studies, we had no directional hypothesis regarding potential interaction effects between negative life events and parental separation.

## Method

### Design and procedure

Data stem from the youth@hordaland (y@h) study, conducted in former Hordaland County in Western Norway in 2012. The y@h study was a population-based study of adolescents with the main aim of assessing mental health and health service use during adolescence. All adolescents born between 1993 and 1995 were invited to participate (aged 16 to 19), whereby 10,257 agreed, yielding a participation rate of 53% for the entire study. Information about the study was given by email, and one school hour was allocated to complete the survey by answering an electronic questionnaire. A teacher organized the data collection and ensured confidentiality. Information about the study was sent by post to those not in school, and alternative solutions for participation were made for students in hospitals or institutions. The present study is based on a subsample of adolescents who had valid information about parental separation, negative life experiences, sociodemographic characteristics, and mental health outcomes (*N* = 7953).

### Representativeness of the sample

Previous investigations have shown that the grade point average of participants in the youth@hordaland study was approximately equal to national and county-level statistics [22]. However, the proportion of parents with higher education was higher than observed in official statistics, although differences in methodology do not allow for direct comparison by numbers. Similarily, the proportion of adolescents categorized as living in a non-separated two-parent family was slightly higher (70% vs. 63%), when compared to the closest possible age group (15–17 years) presented in official statistics in 2012 [23]. Based on these considerations, we note that our sample was skewed toward higher socioeconomic status but representative regarding academic achievement and fairly well captured the distribution of youth in separated families based on official statistics.

### Measures

**Conduct problems** were measured by the Youth Conduct Disorder (YCD) scale [24]. The YCD consists of eight items measured on a binary scale with the answers “yes” and “no”. YCD is a part of the Diagnostic Interview Schedule for Children Predictive Scales [24]. The coefficient omega for ordinal data [ω_𝑢_] was 0.75.

**Attention deficit hyperactivity disorder (ADHD) symptoms** were measured using the Adult ADHD self-report scale (ASRS) from the World Health Organization [25]. The ASRS consists of 24 items rated on a five-point scale, ranging from “never” to “very often”. Previous studies on the ASRS have shown high construct validity and internal consistency in adolescent populations [26]. The *ω*_*u*_ for ordinal data for the sum score was 0.90.

**Depressive symptoms** were measured by the short version of the Mood and Feelings Questionnaire (SMFQ) [27]. SMFQ consists of thirteen items rated on a 3-point Likert scale (“not true”, “sometimes true”, “true”). The SMFQ have been found to confirm well to a unidimensional measurement scale in a previous study from the youth@hordaland sample [28]. The *ω*_*u*_ for the sum score was 0.92.

**Anxiety symptoms** were assessed by the short version of the Screen for Child Anxiety Related Emotional Disorders (SCARED). The SCARED consists of five items measured on a 3-point Likert scale (“not true”, “sometimes true” and “often true”). This short scale has shown similar psychometric properties as the full 41- item version [29]. The *ω*_*u*_ for the sum score was 0.69.

**Negative life events** were measured by five items detailing whether the adolescents had ever experienced (i) ‘a catastrophe or serious accident’, (ii) ‘violence from an adult’, (iii) ‘witnessed someone you care about being exposed to violence from an adult’ and (iv) ‘unwanted sexual actions. In addition, a fifth item detailed whether the adolescents had experienced ‘death of someone close to you’. In case adolescents had experienced death of someone close, they were asked to specify their relationship with the person(s). Death of a (v) parent/guardian, (vi) sibling, (vii) close friend and (viii) girlfriend/boyfriend were included as separate negative events. A cumulative indices of negative life events were created by adding up the number of positive responses. The index scale was coded into 0,1,2,3, and 4 or more, due to few respondents reporting to have experienced five or more negative life events.

**Perceived economic well-being** was measured by asking adolescents to rate how they perceive their economic well-being compared to others. The 3-point Likert scale was used for rating, i.e., “poorer than others”, “equal to others” or “better than others”.

#### Parental education

Maternal and paternal education were reported separately, using the options “primary school,” “high school vocational,” “high school general,” “college/university less than four years,” “college/university four years or more,” and “do not know.” We combined the two high school alternatives into one category (i.e., “high school”).

**Sex** was derived from the personal identity number in the Norwegian National Population Register.

**Age** was taken through the personal identity number in the National Population Register of Norway. Exact age was calculated using the date of participation in the study.

**Parental separation** was defined according to the adolescents’ answer to the questions: “Do your parents live together?” and “Have your parents divorced or separated?” Adolescents confirming that their biological parents did not live together and that they had divorced or separated were defined as having separated parents.

### Statistical analyses

The distribution of the negative life events between adolescents with and without separated parents was examined using Fisher’s exact test due to low number of expected frequencies in some cells. To visualize the distribution of the index scale of number of negative life events between the two groups, a violin plot with a boxplot embedded inside was created.

A series of regression models were conducted to examine the associations between parental separation and negative life events on mental health outcomes. For each outcome, we first tested a crude model with parental separation as the focal independent variable. Gender was added to this model due to the unbalanced gender distribution across the two groups and the well-known association between gender and mental health problems in adolescence. In the next model, the cumulative index of number of negative life events was added to assess whether it attenuated the association between parental separation and mental health problems. In the third and fourth models, we added perceived economic-wellbeing and parental education, to examine whether the association between parental separation and mental health problems would further attenuate when accounting for socioeconomic differences between the two groups. Finally, we tested an interaction model to assess whether the association between negative life events and mental health was moderated by parental separation. The interaction models were tested both without and with adjustments for sociodemographic characteristics. Linear models are presented, as preliminary analyses did not suggest any strong nonlinear associations between negative life events and the mental health outcomes. However, we also present robustness analyses using negative life events as a factor variable (represented by a vector of dummy coded variables) to account for any nonlinear patterns in the associations. The results of the interaction analyses are presented visually. All data preparations, main analyses, and visualizations were conducted using R version 4.1.3 for Windows [30] using functions from the “tidyverse” package [31]. The reliability for each mental health scale was assessed by the coefficient omega for categorical/ordinal data [32], using the lavaan [33] and semTools [34] R-packages.

## Results

Sociodemographic characteristics by parental separation are presented in Table 1. Among those with separated parents, 59% were female participants. Overall, there was a tendency towards lower parental education and perceived economic well-being among those with separated compared to non-separated parents.

**Table 1:** Sociodemographic characteristics of the sample

	Non-separated (n = 5585)	Separated (n = 2368)	*p*-value
	n (%)	n (%)	
Age (mean (SD))	17.44 (0.84)	17.46 (0.83)	0.422
Sex (Female)	3026 (54.2)	1392 (58.8)	< 0.001
Maternal education			
Primary school	351 (6.3)	219 (9.2)	< 0.001
Secondary school	1760 (31.5)	799 (33.7)	
College/university (< 4 years)	966 (17.3)	297 (12.5)	
College/university (4 + years)	1294 (23.2)	477 (20.1)	
Don’t know	1214 (21.7)	576 (24.3)	
Paternal education			
Primary school	367 (6.6)	240 (10.1)	< 0.001
Secondary school	1985 (35.5)	817 (34.5)	
College/university (< 4 years)	594 (10.6)	181 (7.6)	
College/university (4 + years)	1443 (25.8)	412 (17.4)	
Don’t know	1196 (21.4)	718 (30.3)	
Perceived economic well-being			
Worse than others	208 (3.7)	339 (14.3)	< 0.001
Like most others	3791 (67.9)	1586 (67.0)	
Better than others	1586 (28.4)	443 (18.7)	

*Note.p*-values derived from a Welch’s t-test for continuous variables, and a Chi-squared test for categorical variables

The frequency of experiencing negative life events stratified by parental separation is presented in Table 2. Adolescents with separated parents were more likely to have experienced a serious accident, having been exposed to or witnessed violence from a grownup, and unwanted sexual acts than adolescents with non-separated parents. They were also more likely to having experienced death of a close friend or family member but were not more likely to have experienced death of a girlfriend/boyfriend. Adolescents with separated parents were more likely to experience multiple negative life events than those from non-separated families.

**Table 2:** Frequency of negative life events by parental separation

	Non-separated (n = 5585)	Separated (n = 2368)	*p*
*n* (%) confirming			
Serious accident	982 (16.0)	538 (20.1)	< 0.001
Violence from a grown up	458 (7.5)	429 (16.0)	< 0.001
Witnessed violence from a grown up	900 (14.6)	603 (22.5)	< 0.001
Unwanted sexual acts	296 (4.8)	239 (8.9)	< 0.001
Death of a close friend	518 (8.4)	290 (10.8)	< 0.001
Death of a parent/guardian	47 (0.8)	80 (3.0)	< 0.001
Death of a sibling	73 (1.2)	51 (1.9)	0.012
Death of a girlfriend/boyfriend	20 (0.3)	10 (0.4)	0.877
Accumulated negative life events			< 0.001
0	3936 (64.3)	1332 (50.1)	
1	1417 (23.2)	757 (28.5)	
2	542 (8.9)	361 (13.6)	
3	164 (2.7)	140 (5.3)	
4 or more	58 (0.9)	70 (2.6)	
Mean (SD)	0.52 (0.83)	0.79 (1.00)	< 0.001*

*p*-values derived from Fisher´s exact tests. *p-value derived from a Welch’s independent samples t-test

### Regression results

In the regression analyses, parental separation was associated with significantly more symptoms of ADHD, conduct problems, depression, and anxiety, with similar standardized mean differences [SMDs] across the symptom scores (range 0.15 to 0.19). Accounting for number of negative life events (Model 2) partly attenuated and reduced the strength of the associations by about 0.04 to 0.08 standard deviation units but all associations between parental separation and mental health outcomes remained significant. Further adjustments for perceived economic well-being (Model 3) and parental education (Model 4), slightly attenuated the associations between parental separation and mental health problems. However, all associations between parental separation and mental health problems remained statistically significant in the fully adjusted models. The association between negative life events and mental health problems hardly changed when adjusting for perceived economic-well-being and parental education (See Table 3 for details).

**Table 3:** Associations between parental separation and negative life events with mental health symptom scores

	Model 1	Model 2 (+ NLE)	Model 3 (+ PEW)	Model 4 (+ Education)	Model 1	Model 2 (+ NLE)	Model 3 (+ PEW)	Model 4 (+ Education)
	SMD (95% CI)	SMD (95% CI)	SMD (95% CI)	SMD (95% CI)	SMD (95% CI)	SMD (95% CI)	SMD (95% CI)	SMD (95% CI)
	**ADHD symptoms**				**Conduct problems symptoms**			
Separated	0.17(0.13, 0.22)**	0.11 (0.06, 0.16)**	0.08 (0.03, 0.12)**	0.06 (0.01, 0.11)*	0.16 (0.12, 0.21)**	0.11 (0.07, 0.16)**	0.11 (0.07, 0.16)**	0.11 (0.06, 0.16)**
NLE		0.25 (0.22, 0.27)**	0.24 (0.21, 0.26)**	0.24 (0.21, 0.26)**		0.19 (0.17, 0.22)**	0.19 (0.17, 0.22)**	0.19 (0.16, 0.21)**
Adj. R^2^	0.035	0.081	0.087	0.089	0.014	0.043	0.045	0.046
	**Depression symptoms**				**Anxiety symptoms**			
Separated	0.20 (0.15, 0.24)**	0.12 (0.07, 0.16)**	0.08 (0.03, 0.12)**	0.06 (0.02, 0.11)**	0.15 (0.10, 0.19)**	0.11 (0.06, 0.15)**	0.07 (0.03, 0.12)**	0.06 (0.01, 0.10)*
NLE		0.30 (0.28, 0.32)**	0.29 (0.27, 0.31)**	0.29 (0.26, 0.31)**		0.16 (0.13, 0.18**	0.15 (0.13, 0.17)**	0.14 (0.12, 0.17)**
Adj. R²	0.094	0.164	0.174	0.176	0.108	0.132	0.132	0.132

*Note.* Model 1: Adjusted by gender. Model 2: Model 1 + negative life events (NLE). Model 3; Model 2 + Perceived economic well-being (PEW). Model 4; Model 3 + parental education. SMD = Standardized mean difference. Separated; reference category “non-separated”. * *p* < 0.05, ** *p* < 0.01

**Fig. 1:**
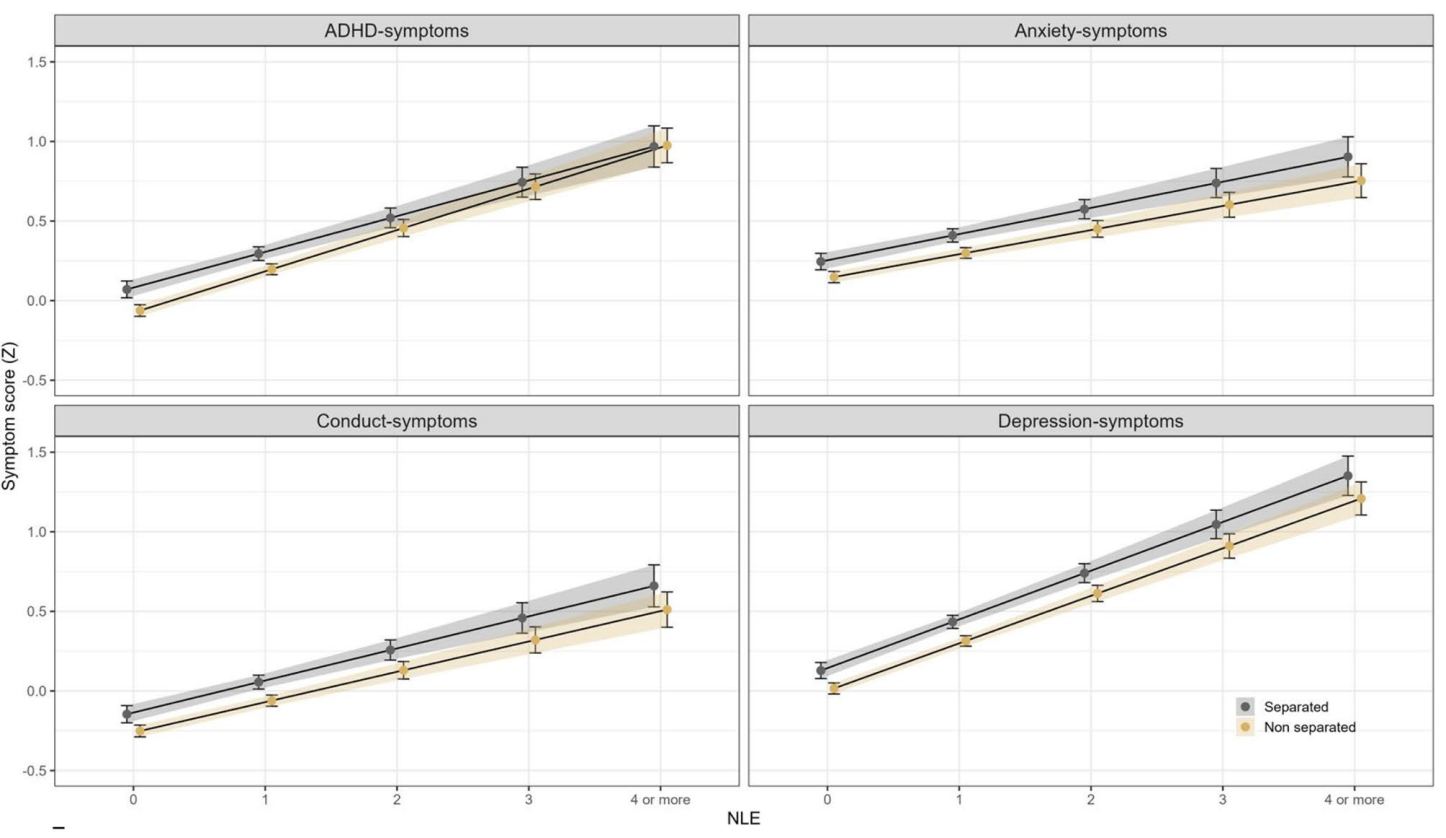
Predicted symptom scores by NLE and parental separation. (Note: This figure shows the predictions from the interaction analyses between parental separation and negative life events (NLE), adjusted by gender (predicted estimates for girls shown). The y-axis is scaled in standard deviation units, where 0 represent the predicted mean of the entire sample. The error bars including the shaded areas represent 95% confidence intervals)

None of the interaction terms between parental separation and negative life events on the mental health outcomes were statistically significant, neither before nor after adjustments for sociodemographic characteristics, suggesting that the slopes of the associations between number of negative life events on all outcomes were similar and not statistically significantly different between adolescents with and without separated parents (see Fig. 1).

Robustness analyses using negative life events as a vector of dummy coded variables to better account for any nonlinear associations yielded a highly similar pattern of results as reported above, both in analysis that adjusted for negative life events and in analysis including an interaction term between parental separation and negative life events (see Supplementary Fig. 1). There were some indications that the association between negative life events and anxiety and depressive symptoms became somewhat stronger from 4 or more negative life events (i.e., a curvilinear relationship). However, we consider this trend uncertain as shown by the wide confidence intervals at 4 or more negative life events.

## Discussion

The aims of the current study were to investigate the distribution of negative life events by parental separation and to assess the direct and interactive association between parental separation and negative life events on adolescents’ mental health problems. For all negative life events except death of a girl/boyfriend, we found that adolescents with separated parents had a higher exposure than peers with non-separated parents. The associations between parental separation and mental health problems were moderately attenuated when adjusting for negative life events, suggesting that negative life events partially accounted for the higher risk of mental health problems among adolescents with separated parents. However, no interaction effects between parental separation and negative life events were detected. Thus, the idea that parental separation amplifies the consequences of negative life events on adolescents’ mental health problems was not supported in the current study.

Adolescents exposed to parental separation had more symptoms of mental health problems relative to their nonexposed peers. The associations were consistent across all mental health outcomes, with adolescents with separated parents scoring about 0.15 to 0.20 standard deviation units higher than nonexposed peers. Overall, these findings align with many previous studies [3, 35, 36], and highlight that adolescents with separated parents are at a higher risk of mental health problems across several domains of mental health. Considering the prevalent occurrence of childhood exposure to parental divorce or separation, the modest effect sizes identified in this study may still be important, as scaled to the population level and across time, they affect many children.

Exposure to negative life events was consistently more common among adolescents with separated parents. For some of these events, such as violence from a grownup and unwanted sexual act, it was twice as likely to have experienced them for adolescents from separated families than their peers with non-separated parents. Adolescents with separated parents were also more likely to have experienced multiple negative life events. Although few studies have focused on more serious negative life events exposure among adolescents with separated parents, our results generally corroborate the findings of existing work suggesting higher exposure of family stress and negative life events among youth with separated parents [5, 6].

When negative life events were accounted for, the strength of the association between parental separation and mental health problems was reduced for about 30 − 40%, depending on the outcome measure. Although we cannot determine whether they experienced these negative life events before or after the parental separation took place, these findings highlight that exposure to multiple negative life events may be one important factor in understanding the association between parental separation and mental health problems in adolescence. Further adjustments of PEW and parental education slightly attenuated the association between parental separation and the mental health outcomes. However, parental separation remained a significant predictor of all mental health problems even after such adjustments, suggesting that socioeconomic differences between those with and without separated parents cannot fully account for the differences in symptom scores. It has been suggested that a parental separation may not only be a cause of future negative life events and stressors but may also act as a source of vulnerability to the effects of such events [17]. We did not find support for this hypothesis, as none of the interaction effects between parental separation and negative life events on adolescents’ mental health problems were statistically significant. Thus, from our results, parental separation and negative life events appear to have more of an additive rather than interactive association with mental health problems in adolescence. These results diverge somewhat from a previous study suggesting that the association between physical abuse and the likelihood of ADHD diagnosis were stronger for adolescents with separated compared to non-separated parents [12]. However, that study focused on a sample of adolescents reported to be abused by the Child Protective services and did not measure cumulative exposure to negative life events, and hence, do not necessarily compare nor generalize to a sample of adolescents drawn from the general population.

### Strengths and limitations

A strength of this study was the large sample size of a well-defined cohort of older adolescents, combined with the assessment of multiple negative life events and mental health problems in adolescents using established measures. The findings should nonetheless be interpreted considering some methodological limitations. First, as many other studies, this study is cross-sectional and we cannot disentangle the direction of the associations between parental separation, negative life events, and mental health outcomes. For example, we cannot establish the degree to which a parental separation leads to higher exposure to negative life events, whether a particular life event was related to the separation of parents, or if these events were already experienced before the separation. Similarly, we cannot ascertain that more mental health problems among youth with separated parents are due to tparental separation or other unmeasured factors may select adolescents into experiencing parental separation and mental health problems. For example, parental mental health problems and interparental conflicts may both increase the risk of parental separation and mental health problems among adolescents [35]. Longitudinal studies tracking these events from before to after the separation are needed to better disentangle the direction of the associations reported in this study. A further potential limitation was the use of self-reported experiences of negative life events, as there is a risk of recollection bias [37]. Thus, some caution should be applied when interpreting the rates of negative life events in the present study. However, we see no reason to assume that recollection bias is more pronounced among adolescents with separated parents compared to their peers with non-separated parents, and that recollection bias would invalidate the relative magnitude of exposure to such events between the two groups, or their associations to mental health outcomes. Moreover, although our measure of negative life events consisted of a parsimonious set of serious events that adolescents may be exposed to, no follow-up information regarding the adolescents´ perception of the severity of the events was collected, which could have further nuanced our findings. Moreover, we cannot exclude the possibility that a more exhaustive measure, also including less severe events, could have yielded different findings. Finally, with a response rate of 53% for the entire study and that the sample size was further reduced due to item non-response, caution should be applied when generalizing the results to the population level.

## Conclusions

Our results demonstrate that adolescents with separated parents are more exposed to negative life events than peers with non-separated parents, and that this added burden may contribute to the observed association between parental separation and mental health problems in adolescence. However, parental separation does not appear to increase the vulnerability to mental health problems following exposure to negative life events among older adolescents.

### Implications and contributions

Given the robust association between negative life events and mental health problems in adolescence, our results highlight the importance of assessing exposure to such events when providing mental health services to adolescents, and particularly when giving services to adolescents with separated parents.

### Electronic supplementary material

Below is the link to the electronic supplementary material.

Supplementary Material 1

## Data Availability

The Norwegian Health research legislation and the Norwegian Ethics committees require explicit consent from the participants to transfer health research data outside of Norway. For the Bergen Child study, which constitutes the data for the current analyses, ethics approval was also contingent on storing the research data on secure storage facilities located in our research institution, which prevents us from providing the data as supplementary information or to transfer it to data repositories. Individual requests for data access should be sent to bib@norceresearch.no contacting the last author of this paper.
