# Supplementary material for: Parental separation, negative life events and mental health problems in adolescence
Source: BMC Public Health. 2023 Nov 29;23:2364. doi: 10.1186/s12889-023-17307-x (PMC10685480; doi:10.1186/s12889-023-17307-x)
Supplement: Supplementary file 1 — Supplementary Material 1 [file 12889_2023_17307_MOESM1_ESM.pdf]

Supplementary Figure 1. Robustness analyses using the index of number of negative life events (NLE) as a factor variable.

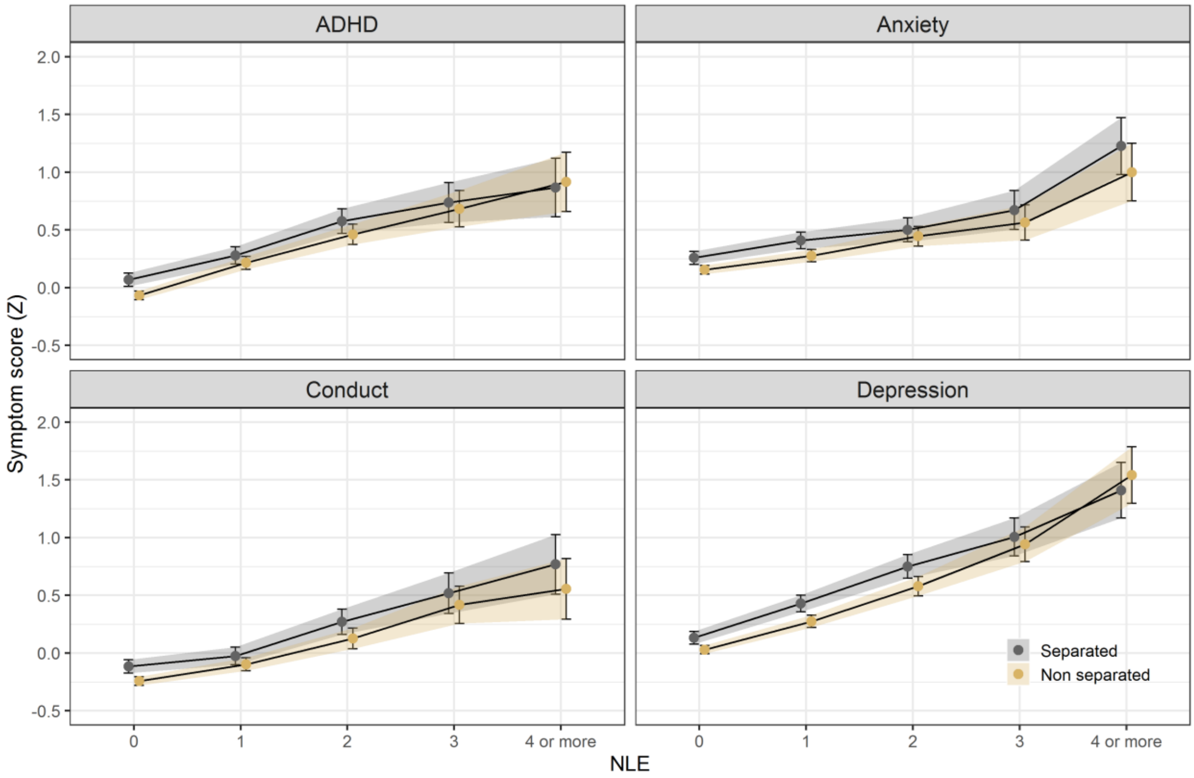

Note. This figure shows the results from the robustness analyses using negative life events as a vector of dummy coded variables to better account for any nonlinear associations.
